# Polyphenolic Profiling and Antioxidant Activity in Berry Extracts of *Pyracantha* Wild Varieties from the Mediterranean Region

**DOI:** 10.3390/antiox13070765

**Published:** 2024-06-25

**Authors:** Roberta Del Sole, Maria Assunta Montefusco, Raffaella Filippini, Lucia Mergola

**Affiliations:** 1 Department of Engineering for Innovation, University of Salento, Via per Monteroni Km 1, 73100 Lecce, Italy; lucia.mergola@unisalento.it; 2 Department of Pharmaceutical and Pharmacological Sciences, University of Padova, Via Marzolo 5, 35131 Padova, Italy; mariasmont@hotmail.com (M.A.M.); raffaella.filippini@unipd.it (R.F.)

**Keywords:** *Pyracantha coccinea*, *Pyracantha angustifolia*, methanol extraction, aqueous methanol extraction, phenolic compounds, antioxidant activity, anthocyanins

## Abstract

*Pyracantha* is a genus of wild perennial shrubs native in an area extending from Southwest Europe to Southeast Asia, and it is used in traditional medicine for the diuretic, cardiac, and tonic properties of its fruits, which can also be cooked to make jellies, jams, and sauces. This work aims to study and compare the antioxidant activity and the phenolic and anthocyanin composition of three varieties of *Pyracantha coccinea*: Red Column (PCR), Orange Glow (PCO), and Soleil d’Or (PCS), and one of *Pyracantha angustifolia*: Orange glow (PAO), collected from the spontaneous flora of the Mediterranean region (Southern Italy). Two different extraction processes were tested using methanol and an aqueous methanol solution (80% MeOH) to evaluate the polyphenolic content and antioxidant activity of freeze-dried berries. The highest total phenolic content was found in PCR and PAO berries (174.21 ± 0.149 and 168.01 ± 0.691 mg of gallic acid equivalent per gram of dry matter, respectively) extracted with an aqueous methanol solution (80% MeOH). Polyphenolic extracts analyzed by HPLC-DAD-ESI/MS confirmed the presence of rutin, quercetin hexose, neoeriocitrin, procyanidin B, and resveratrol. Moreover, the total antioxidant activity of the berries’ extracts was measured by comparing two different spectrophotometric methods (ABTS and DPPH), showing that the varieties with the highest total phenolic content, PCR and PAO, also had the highest scavenging activity. Finally, a suitable extraction process was chosen for the evaluation of the anthocyanins’ composition of all frozen berries, and in all MS spectra of *Pyracantha* varieties, two ionic species at 449 m/z attributable to two cyanidin derivatives were found.

## 1. Introduction

Oxidative stress has been implicated in a wide variety of degenerative processes, diseases, and syndromes. Oxidative damage caused by reactive oxygen species (ROS) on biomolecules, such as lipids, proteins, carbohydrates, and nucleic acids, contributes to the accumulation of injury to cellular constituents [1,2,3,4,5,6]. Since oxidative stress is among the most important reasons causing the formation of neurodegeneration and the use of drugs reduces the symptoms of these diseases, slowing down the progression of disease, but they cannot stop it completely, new and alternative molecules for the treatment of these diseases are also being explored today, such as natural antioxidants occurring in foods and plants, which have gained considerable attention for their few side effects [7,8,9].

Edible fruits constitute a source of nutrients, such as carbohydrates, vitamins, and minerals, as well as non-nutrients, especially polyphenols, which display various health-promoting properties. Polyphenols, which include phenolic acids, flavonoids, stilbenes, coumarins, tannins, and anthocyanins, are widespread secondary metabolites that are found in plants with the function to protect them from biotic and abiotic stresses caused by UV rays, fungal attack, heat exposure parasites, and other pathogens [10,11,12]. Plants and plant parts rich in natural antioxidants were proven to have strong protective activity against the destructive effects of oxidative stress [13,14,15]. The presence of polyphenolic compounds varies in the different parts of the fruit, such as the peel, seed, pulp, or edible part. Moreover, polyphenol distribution in fruits is affected by many factors, such as crop-growing conditions, climate, cultivars, geography, and maturing stage [16,17].

In view of these important properties, in recent years, less popular fruits were also analyzed in order to evaluate the availability of biologically active phytochemicals. Therefore, fruits that display a particularly high antioxidative potential owing to such phytochemicals as proanthocyanidins, anthocyanins, and stilbenes, which are known for their multiple biological activities, have recently gained increasing appreciation. *Pyracantha* or Firethorn is a genus of evergreen shrubs (10 species in the genus), in the Rosaceae family, native in an area extending from Southwest Europe to Southeast Asia. Its distribution is associated with the climate, because *Pyracantha* prefers warm and tender temperatures. For this reason, it is also found in Central and Southern Italy, such as in Salento, an area of Southern Apulia where the Mediterranean climate favors the presence of important varieties of fruits and shrubs. This plant, usually cultivated as an ornamental plant, is also used in traditional medicine for diuretic, cardiac, and tonic properties of its fruits [18,19,20,21]: small bright red or orange berries mature from September to February. The fruits can be cooked to make jellies, jams, and sauces. Very recent reviews on the *Pyracantha* genus demonstrated the growing scientific interest in this plant for its edible and medical value [22,23]. In the last decades, various phytochemical studies of *Pyracantha* plants appeared in the literature and, among them, phenolic studies concerning mainly some species, such as *fortuneana* (or Chinese Firethorn), *coccinea*, and *crenulata* species. Fruits of *P. fortuneana* were studied and active compounds, such as anthocyanins or dibenzofurans and their derivatives, were isolated [24,25,26]. Zhao et al. [27] developed a multilateral assessment process to maximize the in vitro antioxidant capacity, recovery, and bio-accessibility in *P. fortuneana* products. Wang and co-workers reported a study on optimizing the solvent extraction process of antioxidants and α-glucosidase inhibitors from *P. fortuneana* fruits, and some of the chemical components were characterized by HPLC-QTOF [19]. Some studies on *P. coccinea* species have also been reported in the literature. Fico et al. [20] studied the qualitative flavonoid content, while Sarikurkcu and Tepe [28] evaluated the biological activity and phytochemical composition of Turkish *P. coccinea* fruits, and Sahin et al. [29] used response surface methods to optimize the extraction parameters of quercetin and cyanidin and evaluated their reactivity toward reactive oxygen species. However, there are no reports regarding the antioxidant potential and polyphenol content of *Pyracantha* fruits typical of spontaneous flora of Southern Italy, in Salento, also comparing different varieties or species of *Pyracantha*.

**Figure 1:**
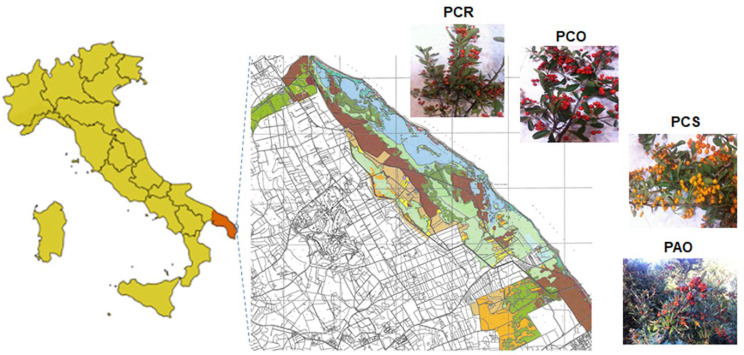
Map of the area (Alimini Lakes) where wild *Pyracantha* plants of *P. coccinea* Red Column (PCR), Orange Glow (PCO), and Soleil d’Or (PCS), and *P. angustifolia* Orange Glow (PAO) were collected.

In the present work, three varieties of *P. coccinea*: Red Column (PCR), Orange Glow (PCO), and Soleil d’Or (PCS), and one of *Pyracantha angustifolia*: Orange glow (PAO), which grow spontaneously in the Mediterranean scrub, were collected from a protected area of Salento (Alimini Lakes), in Apulia (Southern Italy; see Figure 1). Two different extraction solvents were used for phenols’ extraction from the fruits: methanol and an aqueous methanol solution (80% MeOH), and their influence on the extract polyphenolic profile and antioxidant activity was considered, and the results were compared. In detail, total phenolic (TP) content was determined by using the Folin–Ciocalteu method, while total antioxidant activity was assessed by using two spectrophotometric methods: 2,2′-azinobis-(3-ethylbenzothiazoline-6-sulfonic acid) (ABTS)/Trolox equivalent antioxidant capacity (TEAC) and 2,2-diphenyl-1-picrylhydrazyl (DPPH^·^) assays. Moreover, a chromatographic profile of the polyphenols and anthocyanins present in each variety was obtained, and each compound was quantified and identified through HPLC-DAD-ESI-MS.

## 2. Materials and Methods

### 2.1. Chemicals and Reagents

Formic acid (98–100%), sodium sulphate anhydrous (Na_2_SO_4_) (99–100.5%), Trolox (6-hydroxy-2,5,7,8-tetramethylchroman-2-carboxylic acid) (97%), gallic acid (C_6_H_2_(OH)_3_COOH), DPPH (99%), 2,2′-azino-bis(3-ethylbenzothiazoline-6-sulfonic acid) diammonium salt (ABTS) (99%), ethyl acetate (CH_3_COOC_2_H_5_) (99.8%), sodium carbonate (Na_2_CO_3_) (99%), sodium persulphate (Na_2_S_2_O_8_) (≥99%), potassium permanganate (KMnO_4_) (≥99%), and potassium persulphate (K_2_S_2_O_8_) (99%) were purchased from Sigma-Aldrich (Steinheim, Germany). Hydrochloric acid (HCl) (36–38%) was provided by J.T. Baker (Deventer, Holland). Acetonitrile (ACN), water of LC-MS grade, and methanol (MeOH) of analytical grade were acquired from VWR International S.A.S. (Fontenay-sous-Bois, France). Cyanidin-3-O-glucoside was purchased from Extrasynthese (Genay, France). Folin–Ciocalteu’s phenol reagent was acquired from Merck KGaA (Darmstadt, Germany). Deionized water was used to prepare all solutions. C-18 Sep-Pak cartridges and 0.45 μm polypropylene filters were obtained from Waters Corp. (Milford, MA, USA).

### 2.2. Apparatus

Deionized water was provided by a water purification system (Human Corporation, Seoul, Republic of Korea). A rocking-table-type Rotamax 120 from Heidolph Instruments (Schwabach, Germany) was used for shaking. Sonication was carried out using a Sonorex RK 102H ultrasonic water bath from Bandelin Electronic (Berlin, Germany). Centrifugation was achieved with a PK121 multispeed centrifuge from Thermo Electron Corporation (Château Gontier, France). Spectrophotometric studies were carried out using a Jasco V-660 UV-visible spectrophotometer (Jasco, Palo Alto, CA, USA). HPLC analysis was performed using an Agilent 1200 series system (Agilent Technologies, Waldbronn, Germany) consisting of a vacuum degasser, a binary pump, a thermostated autosampler, a thermostated column compartment, and a diode array detector. The instrument was equipped with a 250 mm × 4.6 mm Gemini-NX 5 μm C18 column (Phenomenex, Torrance, CA, USA) set at 32 °C. HPLC-MS analysis was performed using a 6540 quadrupole-time-of-flight (Q-TOF) mass analyzer (Agilent Technologies, Palo Alto, CA, USA) equipped with an electrospray ionization (ESI) source and an ion trap mass analyzer. The chromatograms were recorded using Agilent Mass Hunter software (rev. B.06.00).

### 2.3. Pyracantha Berries Samples

Four different berry varieties of *Pyracantha*: *P. coccinea* M. Roem Red Column, *P. coccinea* M. Roem Orange Glow, *P. coccinea* M. Roem Soleil d’Or, and *P. angustifolia* C.K. Schneid Orange Glow, were hand-harvested in Mediterranean scrub of the protected area of Alimini Lakes, Otranto (Lecce), Italy, in January (Figure 1). After the selection and removal of impurities, the samples were immediately placed in polyethylene bags and stored at −20 °C prior to use.

### 2.4. Preparation of Phenolic Extracts

Frozen fruits were firstly freeze-dried and then 1 g of the powder was macerated with 20 mL of MeOH or aqueous methanol solution (80% MeOH) at 20 °C, overnight in dark shaking conditions. Then, the supernatant was separated by centrifugation at 8000 rpm for 10 min. The precipitate was again extracted with 20 mL of solvent by stirring for 2 h and then it was centrifuged by using the same conditions described above. Finally, the two supernatants were collected, and the solution was filtered through a polypropylene filter (0.45 μm) to remove suspended materials. The extracts were stored at −20 °C for later analysis.

### 2.5. Total Phenolic Content Studies

The total phenolic (TP) content was calculated using a modified Folin–Ciocalteu method [30] with gallic acid as a standard. Then, 0.125 mL of each extract, diluted (1/5, *v*/*v*) with MeOH or 80% MeOH, was mixed with 0.5 mL of water. The sample solutions were added to the Folin–Ciocalteu reagent (0.125 mL) and it was left to react at 25 °C for 6 min, and then NaHCO_3_ was added. Next, 7% Na_2_CO_3_ was used to neutralize the mixture. The absorbance of each solution was measured at 760 nm after incubation for 120 min under stirring (200 rpm) in the dark and at room temperature. The standard curves (R^2^ = 0.995 for the sample prepared with MeOH and R^2^ = 0.999 for the sample prepared with 80% MeOH), obtained using gallic acid solutions at known concentration ranging from 0 to 600 mg/L, were used for calibration. TP content was indicated as the mean of mg of gallic acid equivalents per gram of dry matter (mg GAE/g DM) ± SD for triplicates.

### 2.6. Assay of Antioxidant Activity

The antioxidant activity of all extracts was expressed as Trolox equivalent (TE). In the ABTS assay, also known as the TEAC assay, the green–blue, stable, radical cationic chromophore, 2,2-azinobis-(3-ethylbenzothiazoline-6-sulfonate) (*ABTS*^•+^), is produced by oxidation. Radical *ABTS*^•+^ was generated through oxidation of ABTS by K_2_S_2_O_8_. In particular, a mixture (1:1; *v*/*v*) of K_2_S_2_O_8_ (140 mM) and ABTS solution (7 mM) was prepared, and it was left in the dark for 12–15 h, on an oscillating plate (200 rpm), before use in order to favor the ABTS radical formation. Then, the solution was diluted with MeOH (1/67, *v*/*v*) in order to obtain an absorbance of 0.700 ± 0.100 units at 734 nm. The sample extracts or Trolox standards (20 μL) were combined with the *ABTS*^•+^ working solution (1.980 mL) and allowed to react in dark conditions. After 2.5 min of incubation, the decrease in absorbance at 734 nm was measured. The extent of decolorization was calculated as the percentage reduction of absorbance. The scavenging capability of *ABTS*^•+^ radical was calculated using the following equation:

(1) $${ABTS}^{•+}scavenging\;effect\;\left(\%\right)=\left[1-\frac{A_{A}}{A_{B}}\right]\times 100$$

where, *A_B_* is the absorbance of the initial concentration of *ABTS*^•+^ and *A_A_* is the absorbance of the remaining concentration of *ABTS*^•+^ in the presence of the extract or Trolox. Two standard linear curves of Trolox (in MeOH and 80% MeOH), ranging from 2.5 to 15 μM, were constructed. The results were expressed as μM Trolox equivalents (μM TE).

The *DPPH* scavenging activity assay was carried out according to a method reported by Brand-Williams et al. [31]. The assay procedure was similar to the ABTS method described above. The solution of radical DPPH^•^ (65 μM) was diluted with MeOH or 80% MeOH in order to obtain an absorbance of 0.700 ± 0.100 units at 517 nm. A volume of 20 μL of fruit extracts or Trolox was allowed to react with 1.980 mL of DPPH^•^ radical solution for 15–30 min in the dark, and the absorbance decrease of the resulting solution was monitored. The standard curves were linear between 0 and 15 μM Trolox. The DPPH^•^ radical scavenging activity was calculated using the following equation:

(2) $${DPPH}^{•\;}scavenging\;effect\;\left(\%\right)=\left[1-\frac{A_{A}}{A_{B}}\right]\times 100$$

where, *A_B_* is the absorbance of the initial concentration of DPPH^•^ and*A_A_* is the absorbance of the remaining concentration of DPPH^•^ in the presence of the extract or Trolox. The extract activity was estimated at a minimum of three different concentrations. Similarly, the results were indicated as μM Trolox equivalents (μM TE).

### 2.7. HPLC Analysis for Polyphenols’ Characterization

Phenolic compounds, present in the MeOH extracts of *Pyracantha* samples, were profiled using HPLC-DAD-ESI-MS. The elution was made with 0.1% of formic acid in water (solvent A) and 0.1% of formic acid in ACN (solvent B) using the following gradient: 0 min, 7% B; 3 min, 15% B; 20 min, 35% B; 45 min, 70% B; 120 min, 100% B; 140 min, 100% B. The column was thermostated at 32 °C using a flow rate of 0.3 mL/min and an injection volume of 10 μL. Absorbance spectra were acquired every 2 s in a range of 190–590 nm (4 nm of bandwidth), and each chromatogram was recorded at 280 nm. The injections were performed in triplicate for each sample.

ESI-MS was performed to determine the molecular formulas of chromatogram peaks. The mass analysis was performed using a Q-TOF mass analyzer with an electrospray ionization source (ESI). A positive ion mode was chosen for ionization and the mass spectra were registered between 100 and 1600 *m*/*z* using the following instrumental conditions: capillary voltage of 4000 V, drying gas temperature of 350 °C, fragmentor potential of 150 V, drying gas flow rate of 12.0 L/min, spray voltage of 5000 V, nebulizer gas pressure of 45 psi, and skimmer voltage of 45 V.

### 2.8. Quantification of Polyphenols in the Pyracantha Extracts

Quantification of polyphenols in *Pyracantha* varieties was carried out with an external standard method. A series of diluted solutions were prepared from a standard solution of gallic acid, and the corresponding calibration curve was used for quantification of absorbance peaks observed in HPLC-DAD chromatograms. Gallic acid was solubilized in aqueous 0.01% HCl (*v*/*v*), and five concentrations (0.1, 0.2, 1, 5, and 10 μg/mL) were prepared and used to obtain a linear calibration curve with R^2^ = 1. The concentration of each polyphenol was determined by using the peak area of the chromatogram acquired at 280 nm against the calibration curve constructed from gallic acid standards. Concentrations were expressed as the mean of mg of polyphenols per 100 g of dry matter (mg/100 g DM) ± SD for triplicates.

### 2.9. Anthocyanins’ Extraction and Purification

Acidic conditions were used for anthocyanins’ extraction [32]. In particular, 4 g of frozen berries (the entire fruit was used with skin and seeds) were first crushed and then extracted using 50 mL of 0.1% HCl (*v*/*v*) in MeOH in the dark, for 24 h at room temperature. The mixture was filtered, and the residual solid was washed several times until a clear solution was obtained. After filtration, all solutions were combined and evaporated at room temperature. The residual solid was dissolved in 6 mL of 0.01% aqueous HCl (*v*/*v*) and then purified using a C-18 Sep-Pak cartridge (1 g). To activate the cartridge, MeOH and 0.01% aqueous HCl (*v*/*v*) were used. After sample loading, the cartridge was washed several times with 0.01% aqueous HCl (*v*/*v*) to remove sugars, acids, and other water-soluble compounds. Subsequently, less polar polyphenols were eluted using ethyl acetate. Finally, in order to elute anthocyanins derivatives, 0.01% HCl (*v*/*v*) in MeOH was used, until the discoloration of the solution. All eluates were combined, evaporated at room temperature, and then dissolved in 0.01% aqueous HCl (*v*/*v*) at a known concentration (6 mg/mL). All procedures were conducted in the dark to prevent oxidation processes.

### 2.10. Anthocyanins’ Separation and Characterization

HPLC-DAD-ESI-MS was used in order to separate all anthocyanins present in *Pyracantha* extracts. Here, 0.1% formic acid in ultrapure water (phase A) and 0.1% formic acid in ACN (phase B) were used as mobile phases, and the following elution program was used: 0 min, 93% A and 7% B; 3 min, 85% A and 15% B; 30 min, 65%A and 35% B; 65 min, 30% A and 70% B; 70 min, 70% A and 7% B. The column was thermostated at 32 °C with a flow rate of 0.3 mL/min and an injection volume of 10 µL. All spectra were acquired in the range of 190–590 nm, with a bandwidth of 4 nm, and the chromatograms were registered at 520 nm. The injections were performed in triplicate for each sample. Anthocyanins’ identification was conducted using the same MS experimental condition mentioned above. Finally, anthocyanins were quantified using an external standard (cyanidin-3-glucoside chloride). Six concentrations of 0.25, 1, 5, 10, 25, and 50 µg/mL were used to prepare a linear calibration curve, and anthocyanins’ quantification was calculated using the peak area of the compound identified in the chromatogram acquired at 520 nm. Anthocyanins’ concentration was indicated as the mean of mg of anthocyanins per 100 g of frozen matter (mg/100 g FM) ± SD for triplicates.

### 2.11. Statistical Analysis

All experiments were performed in triplicate. Data were shown as mean ± SD. The correlation analysis results of TP content versus ABTS and DPPH scavenging activity were shown as Pearson correlation coefficients (PCC) and *p*-value using MS Excel 2021 from Microsoft (Redmond, WA, USA) [33].

## 3. Results

### 3.1. Extraction of Polyphenols in Pyracantha Varieties

The nature of the organic solvent used during the extraction process is important to improve the amounts of extracted polyphenols. For this reason, a study using two different extraction conditions was conducted. Since the presence of water in combination with organic solvents forms a relative polar medium, improving the phenolic compounds’ extraction, in this work, two different solvent extractions were considered. In particular, fruits of four *Pyracantha* varieties (PCR, PCO, PCS, and PAO) were lyophilized in order to improve process extraction from peel and pulp, and then subjected to extraction with MeOH, a common solvent used for polyphenols’ extraction, and an aqueous methanol solution (80% MeOH).

TP content of all extracts was evaluated by the Folin–Ciocalteu colorimetric test using gallic acid as a reference standard.

**Figure 2:**
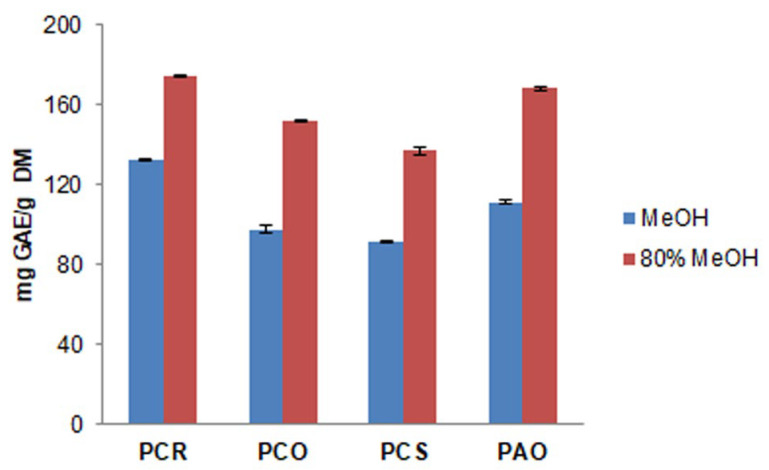
Comparison of total phenolic (TP) content of extracts obtained using MeOH and 80% MeOH as extraction solvents.

In Figure 2, the TP content of *Pyracantha* varieties extracted with MeOH and 80% MeOH is compared. The results revealed a significant influence of water in the extraction process, with a content of polyphenols extracted that was 20–30% higher than the polyphenolic content of extracts obtained with MeOH. In both MeOH and 80% MeOH, the same trend of polyphenolic content was found in the fruits of the different varieties: PCR > PCO > PCS. Moreover, PCR and PAO species showed the highest polyphenolic content using both extraction solvents. TP contents of 174.21 mg GAE/g DM ± 0.149 were found in PCR and 168.01 mg GAE/g DM ± 0.691 in PAO when the extraction process was carried out with 80% MeOH.

Recently, Wang and co-workers evaluated the TP content of *P. fortuneana* fruits present in China using different solvents for extraction (acetone, ethanol, MeOH, and aqueous methanol solution) [19]. In our work, the TP content obtained from different varieties of *P. coccinea* and *P. angustifolia* (Figure 2) was much higher than the results reported for *P. fortuneana* (Chinese), which showed values between 7.78 and 17.33 mg GAE/g DM [19]. The TP content in MeOH extracts of all *Pyracantha* species studied in the current work was in the range of 91.42–132.57 mg GAE/g DM, which was higher than the polyphenolic content of Chinese *P. fortuneana* (about 37 mg GAE/g DM) studied by Zhao and co-workers [27]. These differences can be associated with the different extraction processes (ultrasonic bath, contact time, and temperature around 50 °C). In our work, the extraction was performed at 20 °C, overnight, in the dark under shaking conditions in order to increase the contact time of the solvent with the freeze-dried fruit powder, maximizing the polyphenolic extraction and preserving the degradation of polyphenols caused from an excess of temperature. Indeed, it is well known that high temperatures can lead to a degradation of antioxidant compounds. However, the high value of the TP can also be related to the specificity of the berries studied.

### 3.2. In Vitro Assay of Total Antioxidants

As previously reported, quantitative analysis obtained in terms of TP content showed a significant difference between the considered samples, and in this section we focused our attention on the corresponding antioxidant activity of the extracts.

It is difficult to identify a single method of analysis that can unambiguously measure the ability of a compound to counteract the oxidative action of reactive species, especially in complex matrices, such as plants or extracts. Indeed, due to the complex reactivity of the phytoconstituents, the antioxidant activity of plant extracts and food products cannot be assessed by a single method, but the use of at least two different tests for the determination of the antioxidant activity is recommended. For this reason, the antioxidant activity of the samples was determined by two spectrophotometric methods, ABTS and DPPH, and expressed in Trolox equivalents.

**Figure 3:**
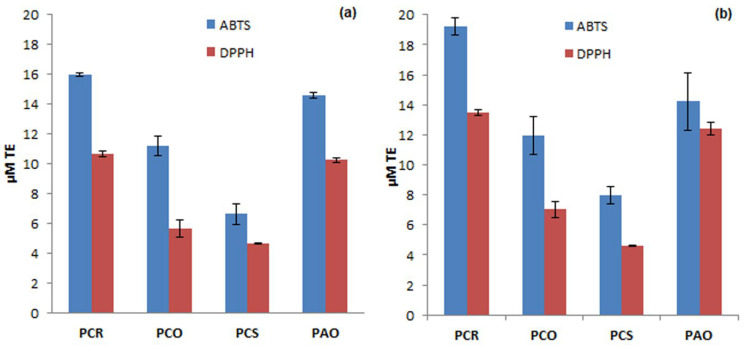
Comparison between µM TE values of MeOH (**a**) and 80% MeOH (**b**) extracts obtained using ABTS and DPPH methods.

In particular, ABTS and DPPH scavenging activities were evaluated on all extracts of *Pyracantha* varieties (in MeOH and 80% MeOH) considered in this study. After construction of a calibration curve using Trolox as an antioxidant probe in MeOH and 80% MeOH, the percentage reduction of absorbance (I%) at 734 nm was evaluated, and the corresponding concentration, expressed as µM TE, was calculated. In Figure 3, a comparison between μM TE values obtained with both ABTS and DPPH methods of MeOH (a) and 80% MeOH (b) extracts, respectively, is reported.

As it can be seen in Figure 3, all *Pyracantha* extracts exhibited scavenging activity toward both *ABTS*^•+^ and *DPPH*^•+^ radicals, even if significant differences can be noted between the different berry varieties. However, both methodologies showed similar trends for the different varieties, with higher values always obtained using the ABTS method if compared with the scavenging activity obtained using the DPPH method for the same berry. The best results were obtained with PCR and PAO extracts using both ABTS and DPPH methods, while PCS was the least active product. Moreover, extracts prepared using 80% MeOH (b) for solvent extraction (Figure 3) showed better antioxidant activities, demonstrating a highly significant, positive correlation between the studied antioxidant activity and the total phenolic content. Therefore, the measured antioxidant activities are mainly attributable to the phenolic compounds present in the extracts. It can be concluded that the determination of phenolic content provides a good estimate of the antioxidant capacity of the extracts themselves.

**Figure 4:**
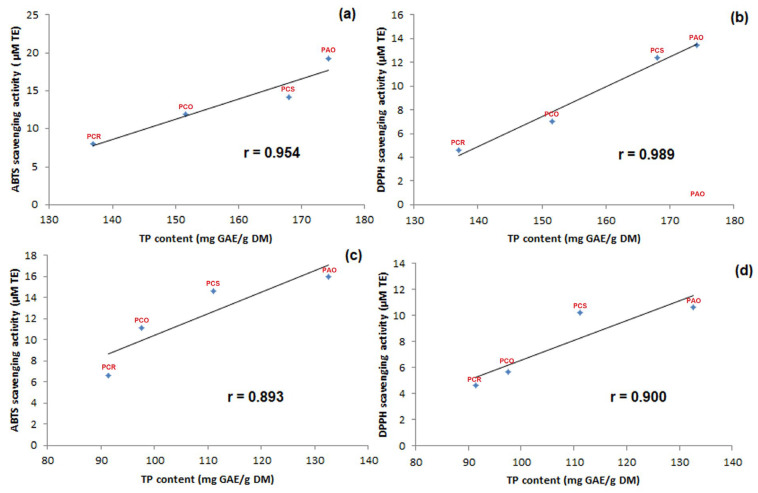
Pearson correlation scatter plot of the relationship between ABTS scavenging activity and TP content in 80% MeOH extracts (**a**). DPPH scavenging activity and TP content in 80% MeOH extracts (**b**). ABTS scavenging activity and TP content in MeOH extracts (**c**). DPPH scavenging activity and TP content in MeOH extracts (**d**).

To corroborate the above findings, statistical analysis was performed between TP contents and DPPH and ABTS scavenging activity, and the results were expressed as the Pearson correlation coefficient (PCC), also known as Pearson’s r (Figure 4). The PCC indicates the direction and the strength of the linear relationship of correlation.

As can be seen in Figure 4a,b, when 80% MeOH extracts were analyzed, the PCC between ABTS scavenging activity and TP content (Figure 4a) was 0.954 (*p*-value < 0.045), representing a strong positive relationship. A similar result was obtained when the other method was evaluated, with a PCC between DPPH scavenging activity and TP content (Figure 4b) equal to 0.989 (*p*-value < 0.010). On the other hand, when MeOH extracts were considered, the PCC between ABTS scavenging activity and TP content (Figure 4c) was 0.893 (*p*-value < 0.107), and the PCC between DPPH scavenging activity and TP content (Figure 4d) was 0.900 (*p*-value < 0.099), representing a weak positive relationship.

According to these results, we can confirm the initial hypothesis that the antioxidant activity was due mainly to the TP content, and both methods yielded similar trends for the different *Pyracantha* varieties studied, with a stronger correlation in 80% MeOH extracts due to the higher TP content found in these extracts compared to the extracts in MeOH.

### 3.3. HPLC Profiling of Phenolic Compounds in MeOH Extracts

**Figure 5:**
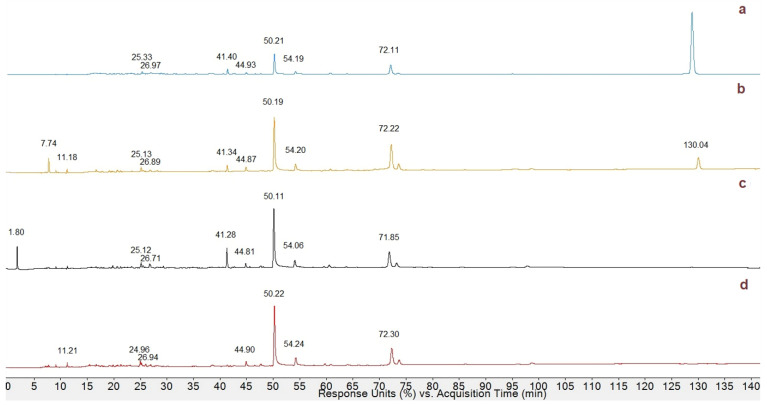
HPLC-DAD chromatograms acquired at 280 nm of the MeOH extracts of PAO (**a**), PCR (**b**), PCO (**c**), and PCS (**d**) varieties.

A study by Zhao et al., carried out on *P. fortuneana* (Chinese), was taken as a reference for the phenolic profile and the antioxidant capacity of berries extracted in MeOH. HPLC-DAD chromatograms of PCR, PCO, PCS, and PAO MeOH extracts (Figure 5) were examined, and the peaks at 280 nm were compared [27].

As it can be seen, *Pyracantha* varieties showed a similar chromatographic profile in the first 75 min, while at around 130 min, a new peak with significant intensity only in PCR and PCO varieties could be observed. In order to propose an identification of each polyphenol, mass spectra associated with literature data [19,27,28] and online research in the polyphenol database http://phenol-explorer.eu (accessed on 20 March 2024) was evaluated.

**Figure 6:**
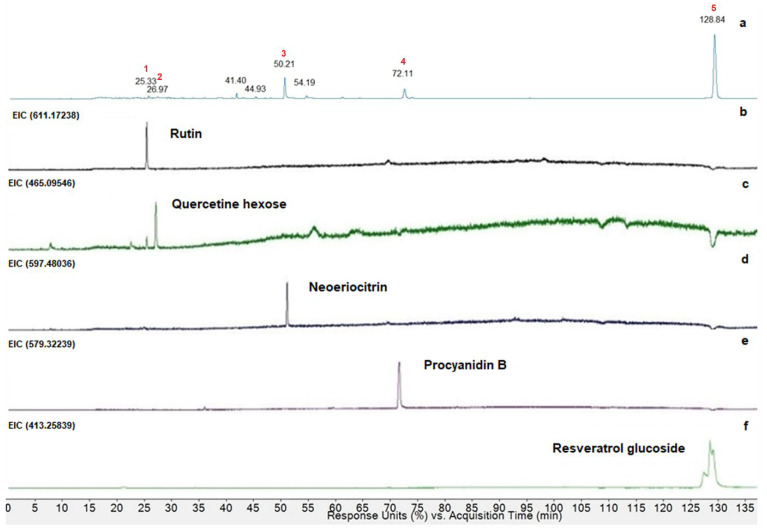
HPLC-DAD chromatograms at 280 nm of PAO MeOH extracts (**a**). EIC at: 611 *m*/*z* of rutin (**b**), 465 *m*/*z* of quercetin-hexose (**c**), 597 *m*/*z* of neoeriocitrin (**d**), 579 *m*/*z* of dimer procyanidin B (**e**), and 413 *m*/*z* of resveratrol glucoside (**f**).

In Figure 6, HPLC-DAD at 280 nm of PAO extract (Figure 6a) was compared with extracted ion chromatograms (EIC) of the same variety subjected to HPLC-ESI-Q-TOF in positive mode.

As reported in Figure 6, five polyphenols were characterized. In particular, the compounds corresponding to peaks 1, 2, 3, 4, and 5 were tentatively identified as rutin, quercetin hexose, neoeriocitrin, dimer procyanidin B, and resveratrol glucoside, comparing the molecular ions (M-H)^+^ or (M-Na)^+^ with the molecular weight of the compounds previously mentioned, and using the polyphenol database and literature data, although the identification of isomers is difficult. In this case, other information, such as the UV spectra or the retention time available in the literature, were considered and crossed with the data obtained. Tentatively identified polyphenols in MeOH extract from PAO are shown in Table 1.

Quantification analysis of the identified compounds was performed using gallic acid as an external standard, and all concentrations were expressed in milligram per 100 g of DM (mg/100 g DM; Table 2).

The results obtained from HPLC-MS analysis of MeOH extracts of *Pyracantha* showed, in all extracts, the presence of four of the identified polyphenols. A significant difference in terms of the concentration of different polyphenols was noted. In particular, neoriocitrin and dimeric procyanidin B were the most abundant polyphenols. Peak 5 (resveratrol glucoside) was found only in PAO (184.89 mg/100g DM) and PCR (10.69 mg/100 g DM) varieties. Moreover, comparing our results with some reference works [19,20,27], the absence of resveratrol glucoside both in *P. coccinea* Red Column grown in the north of Italy and Chinese *P. fortuneana* can be observed. The data obtained showed the highest content of polyphenols in PCR and PAO varieties, confirming the results previously obtained in terms of TP and scavenging activity.

### 3.4. Extraction, Quantification, and Identification of Anthocyanins

In order to extract and identify the anthocyanin content of *Pyracantha* varieties of Salento, a first extraction process was performed using lyophilized berries, but poor results have been obtained, probably for the degradation occurring during dehydration. For this reason, the extraction process was conducted on frozen berries following the classic method of Longo and Vasapollo [32]. Firstly, frozen berries were treated with acidified MeOH, and the extracts obtained were purified on solid-phase extraction cartridges to remove sugars. All eluates were evaporated, and anthocyanin content was expressed as milligrams per 100 g of frozen matter. PCR variety showed the highest extraction yield (293.64 mg/100 g FM), also confirmed from the intense purple coloring of 0.01% HCl (*v*/*v*) aqueous solution during the extraction process, while the *Angustifolia* species yielded the lowest extraction yield (165.48 mg/100g FM), despite the red color of the berry.

**Figure 7:**
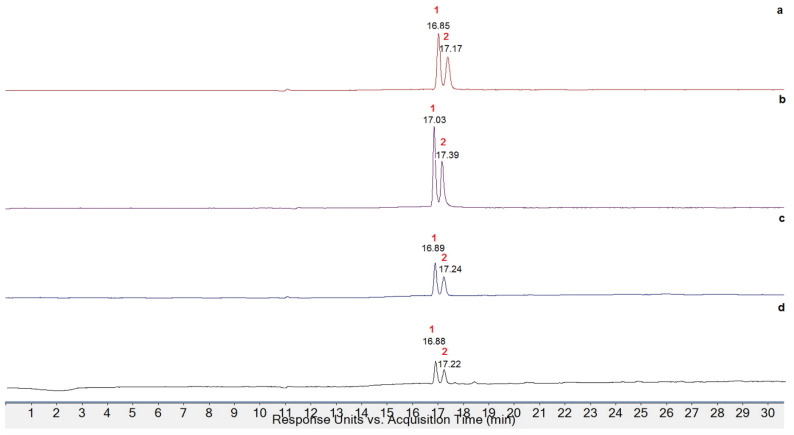
Enlarged HPLC-DAD chromatograms acquired at 520 nm of (**a**) PCR, (**b**) PCO, (**c**) PCS, and (**d**) PAO.

Anthocyanin characterization was performed through HPLC-DAD-ESI-MS, comparing the retention time and mass data with reference standards and literature data. In Figure 7, the enlarged chromatograms obtained at 520 nm for the four *Pyracantha* samples are reported. As can be seen, all extracts showed the same chromatographic profile with the presence of only two peaks, and their mass was determined through HPLC-ESI-MS.

**Figure 8:**
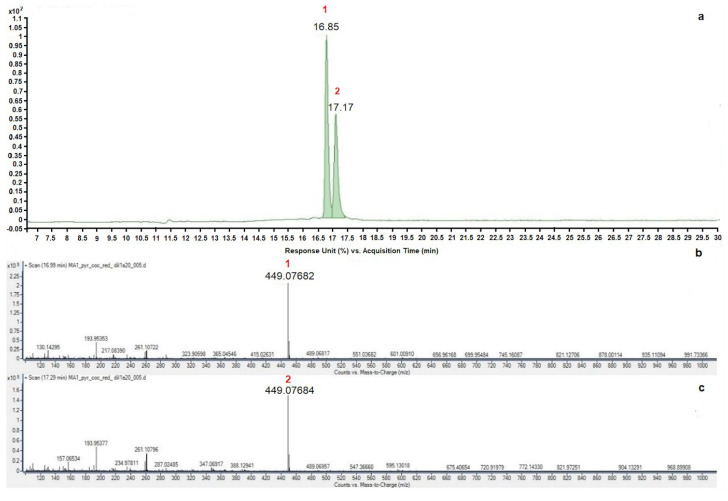
HPLC-DAD chromatogram acquired at 520 nm (**a**) and mass spectra of anthocyanins of peak 1 (**b**) and peak 2 (**c**) relative to the PCR variety and common in all analyzed samples of *Pyracantha*.

In all MS spectra of *Pyracantha* varieties, two ionic species at 449 *m*/*z*, attributable to two cyanidin derivatives, were found. Comparing the retention times with the external standard (cyanidin-3-glucoside chloride) and with literature data [27], it can be supposed that the anthocyanins present in all samples were cyanidin-3-O-glucoside and cyanidin-3-O-galattoside (Figure 8).

**Figure 9:**
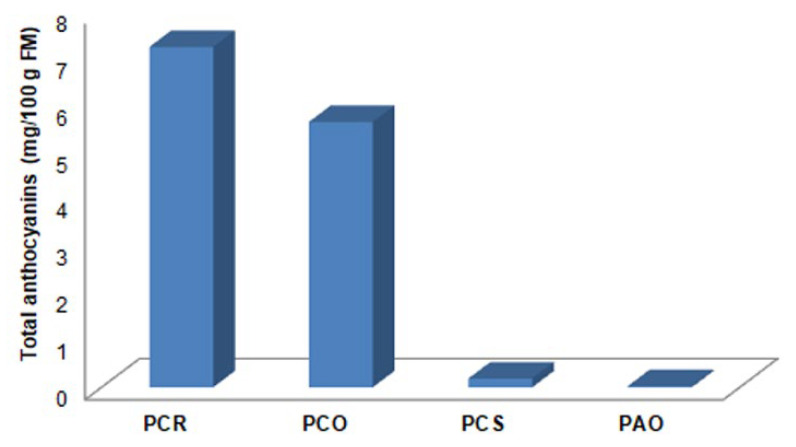
Total anthocyanins content (1 and 2) in all samples of *Pyracantha* analyzed.

Finally, anthocyanins were quantified using an external standard (cyanidin-3-O-glucoside), and the anthocyanin content is reported in Table 3. As it can be seen, PCR showed higher concentrations of both cyanidins found, and the total anthocyanins concentration in each berry (Figure 9) showed higher values in the red *P*. *coccinea* varieties (PCR and PCO) than in the orange one (PCS), and in the red *P*. *angustifolia* variety.

## 4. Conclusions

In this work, an overview on the possible contribution of the phytochemical composition of *Pyracantha* berries of Salento to the biological activity was provided. For the experiments, fully ripe berries were considered since a higher content of bioactive compounds is expected.

A high correlation between the polyphenolic content of the extracts and their antioxidant capacity was observed. Furthermore, it has been seen that the presence of water in the solvent extraction (80% MeOH) enhanced the polyphenolic content of the extracts and their scavenging activity compared to MeOH extracts.

Phytochemical analysis revealed the presence of different compounds, such as cyanidin derivatives, resveratrol, quercetin, neoriocitrin, rutin, and procyanidin dimer-type B, which are certainly responsible, at least in part, for the antioxidant activity of the extracts. Comparing the obtained results with literature data of similar analyses conducted on different *Pyracantha* varieties, it can be concluded that *Pyracantha* berries of Salento, in particular, *P. coccinea* M. Roem Red Column (PCR) and *P. angustifolia* (Franch.) C.K. Schneid Orange Glow (PAO), can represent a good source of phenolic antioxidants. The potential therapeutic role and the chemopreventive action in various pathologies of polyphenolic compounds, when consumed as food, should be a strong stimulus for research and valorization of local plant resources, such as *Pyracantha* species.

## Figures and Tables

**Table 1:** Tentatively identified polyphenols in MeOH extract from PAO by HPLC-ESI-Q-TOF.

Peak	t_R_	ʎ_max_	MW	M-H^+^ or M-Na^+^ (*m*/*z*)	Proposed Compound	Classification
1	25.33	280	610	611.17238	Rutin	Flavonol
2	26.97	280	464	465.09546	Quercetine hexose	Flavonol
3	50.21	280	596	597.48036	Neoeriocitrin	Flavanone
4	72.11	280	578	579.32239	Procyanidin B	Tannin
5	128.84	280	390	413.25839	Resveratrol glucoside	Stilbenoid

**Table 2:** Qualitative and quantitative polyphenolic profiles of MeOH *Pyracantha* extracts through HPLC-MS analysis.

	PCR	PCO	PCS	PAO			PCR	PCO	PCS	PAO
	Retention Time (min)				MW (M-H^+^, M-Na^+^)		(mg/100 g DM) ^a^			
1	25.13	25.12	24.96	25.33	610 (611, 633)	Rutin	2.13	2.27	2.48	3.50
2	26.89	26.71	26.94	26.97	464 (465)	Quercetin-hexose	1.33	2.81	1.40	3.40
3	50.19	50.11	50.22	50.21	596 (597)	Neoeriocitrin	29.46	30.29	30.78	33.27
4	72.22	71.85	72.30	72.11	578 (579)	Procyanidin B	20.80	12.47	14.05	23.65
5	130.04	-	-	128.84	390 (391, 413)	Resveratrol glucoside	10.69	-	-	184.89

^a^ Relative standard deviation (RSD) of all identified peaks < 5%.

**Table 3:** Anthocyanins concentration in *Pyracantha* extracts.

Sample	Peak (Retention Time)	Concentration (mg/100 g FM) ^a^
PCR	1 (16.85 min)	4.21
	2 (17.17 min)	3.05
PCO	1 (16.93 min)	3.25
	2 (17.39 min)	2.41
PCS	1 (16.89 min)	0.11
	2 (17.24 min)	0.08
PAO	1 (16.88 min)	0.02
	2 (17.22 min)	0.01

^a^ Relative standard deviation (RSD) of all identified peaks < 5%.

## Data Availability

Data are contained within the article.
